# Colovaginal Fistula: An Uncommon Complication After Vaginal Hysterectomy and Pelvic Floor Repair With Mesh Surgery

**DOI:** 10.7759/cureus.51221

**Published:** 2023-12-28

**Authors:** Yin Ru Tan, Jill Cheng Sim Lee, Sharmini Su A Sivarajah, Cheryl Xi Zi Chong, Hong Liang Chua, Kazila Bhutia

**Affiliations:** 1 Department of Obstetrics & Gynaecology, Kandang Kerbau (KK) Women’s and Children’s Hospital, Singapore, SGP; 2 Department of Urogynaecology, Kandang Kerbau (KK) Women’s and Children’s Hospital, Singapore, SGP; 3 Department of General Surgery, Sengkang General Hospital, Singapore, SGP

**Keywords:** vaginal hysterectomy, mesh, pelvic floor repair, complication, colovaginal fistula

## Abstract

A colovaginal fistula (CVF) is an abnormal epithelialized connection between the colon and the vagina. It is a rare complication following gynaecology surgery and can significantly affect patients’ quality of life. CVFs are commonly associated with diverticular disease and are usually seen in patients with a previous hysterectomy. We report an uncommon case of postsurgical CVF following vaginal hysterectomy with mesh-augmented pelvic floor repair, which was unrelated to diverticulitis. The patient was successfully managed by a multidisciplinary team with staged surgery.

## Introduction

A fistula is an abnormal communication between two epithelialized surfaces and may result from infection, inflammation, trauma, or surgery [1,2]. A colovaginal fistula (CVF) is an abnormal epithelialized connection between the colon and the vagina. Causes of CVFs include diverticular disease, malignancy, radiation therapy, surgery, infection and inflammatory bowel conditions [3]. Although uncommon, CVFs are the third commonest lower reproductive tract fistulas surgically repaired [1].

We report a case of a postsurgical CVF following vaginal hysterectomy with mesh-augmented pelvic floor repair, as well as the subsequent management of the patient. Written informed consent was obtained from the patient. This case was previously presented as a poster at the International Urogynecological Association (IUGA) 48th Annual Meeting on 21 June 2023.

## Case presentation

The patient was a healthy 69-year-old woman with three previous normal vaginal deliveries. Her medical history was unremarkable. She underwent a vaginal hysterectomy, right salpingo-oophorectomy, and anterior mesh-augmented pelvic floor repair with posterior native tissue repair for pelvic organ prolapse. A grade-three cystocele, grade-three uterovaginal descent and grade-two rectocele based on the Baden-Walker Halfway system were noted intraoperatively. The uterus was atrophic, and both tubes and ovaries were normal. Right salpingo-oophorectomy was performed in view of persistent oozing from adhesions to the right fallopian tube. A four-armed type one polypropylene transvaginal mesh kit was used. Cystoscopy performed then was normal. The total estimated blood loss was 200ml. The patient had a low-grade fever of 37.7 degrees Celsius on the third postoperative day with raised inflammatory markers which subsided the following day. Histology of the uterus, cervix, right tube, and ovary was benign.

**Figure 1:**
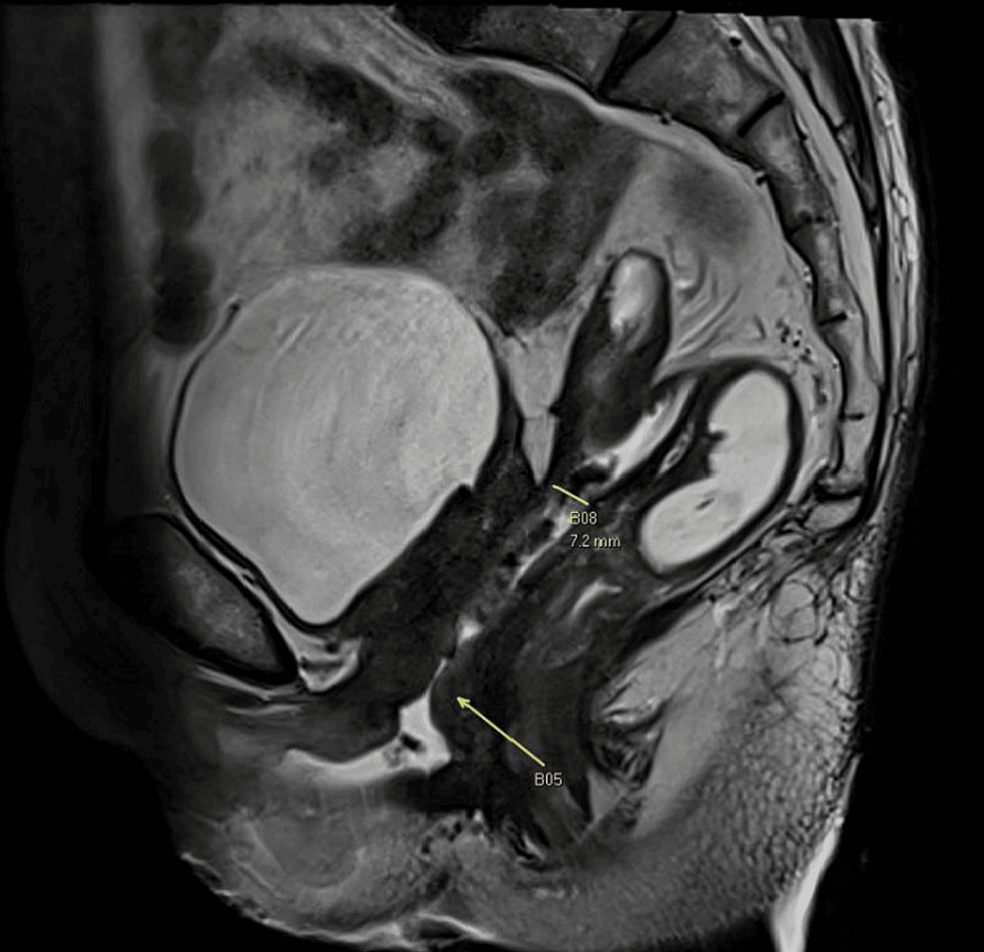
MRI pelvis showing the colovaginal fistula

**Figure 2:**
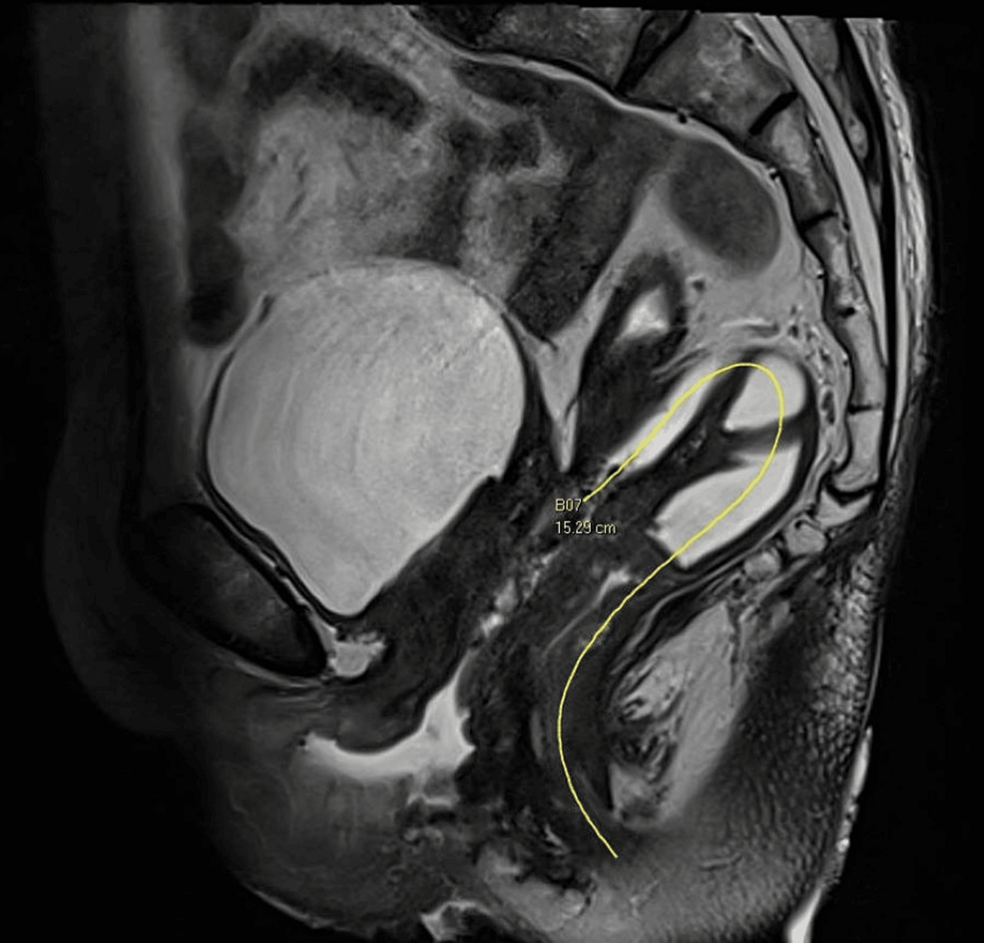
MRI pelvis showing the colovaginal fistula

The patient presented two months postsurgery with symptoms of daily feculent vaginal discharge. A 1cm vaginal vault defect was noted 6cm from the hymen. A pelvic MRI revealed a fistulous communication between the sigmoid colon and vaginal vault, 15.3cm from the anal verge measuring 1.6 x 0.7cm (Figures 1, 2).

There was no abscess, collection or free air. Colonoscopy did not identify any lesions, sutures or mesh, although gas leak was noted transvaginally. There was no evidence of diverticular disease on imaging or colonoscopy. The patient was offered options of surgery or conservative management to await spontaneous closure. She preferred conservative management at this stage. She was reviewed at three-month intervals and initially reported improving symptoms.

One year after her initial surgery, she reported worsening vaginal discharge with perineal irritation. Physical examination revealed exposed mesh measuring 4x4 cm which was loosely hanging from the anterior vaginal wall with the presence of epithelialized vaginal mucosa beneath the mesh. Faeces was noted in the vagina and a 1cm CVF opening was identified at the vault line. She wanted the fistula and mesh removed surgically. Following a multidisciplinary discussion with our team of urogynaecologists and colorectal surgeons, the patient underwent staged surgery comprising: 1) laparoscopic defunctioning colostomy, examination under anaesthesia with partial excision of vaginal mesh and cystoscopy, 2) transvaginal excision of remaining vaginal mesh and cystoscopy, 3) laparoscopic low anterior resection and vaginal vault closure, 4) closure of transverse loop colostomy over the period of seven months.

**Figure 3:**
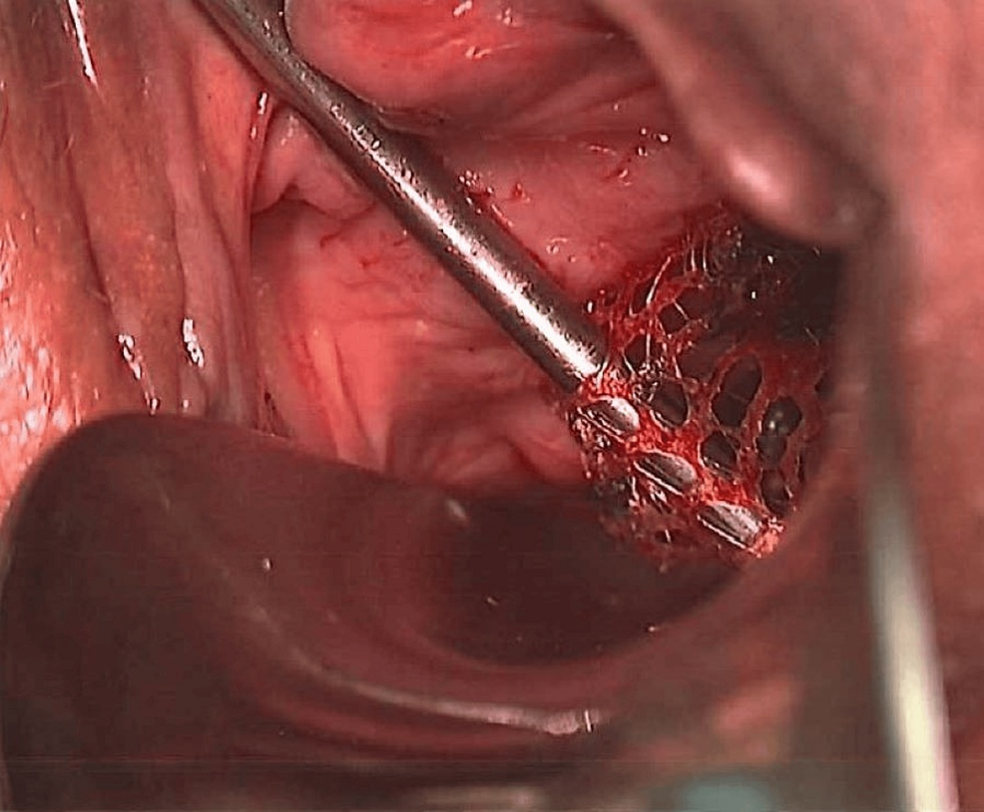
Vaginal mesh extrusion seen intraoperatively

The patient first underwent a laparoscopic defunctioning colostomy, examination under anaesthesia with partial excision of vaginal mesh, and cystoscopy. Intraoperative vaginal examination confirmed a 4x4 cm area of extruded mesh hanging loosely from the anterior vaginal wall, with mesh edges fixed at both lateral vaginal walls (Figure 3).

The vaginal mesh was partially excised in the mid-segment to reduce mesh load to aid in the healing process. The 1cm CVF opening was noted at the centre of the vaginal vault. Cystoscopy performed was normal, with no evidence of mesh in the bladder. Adhesions were noted on laparoscopy, but the CVF was not well visualised laparoscopically.

A month later, the patient underwent transvaginal excision of remaining vaginal mesh and cystoscopy. Intraoperatively, the edges of the mesh were felt on both sides of the anterior vaginal wall (4x1cm on each side). The vaginal mesh was excised via sharp and blunt dissection on both edges up to the lateral fornices. The vaginal defect was closed with Vicryl sutures. A cystoscopy done at the end of the procedure was normal. The patient was reviewed three weeks postoperatively, and no mesh was palpable on vaginal examination.

The patient subsequently underwent laparoscopic low anterior resection and closure of the vaginal vault two months later. The fistula from the sigmoid colon to the vagina was visualised laparoscopically. The descending colon and rectum were mobilised down to the level of the pelvic floor. The rectovaginal septum was dissected and low anterior resection with end-to-end anastomosis was performed. Vaginal vault closure was performed with two-layer closure both laparoscopically and transvaginally with v-loc and vicryl sutures respectively. Cystoscopy done after the procedure was normal. The patient was reviewed four weeks postoperatively in clinics and no fistula or mesh was noted on vaginal examination. She then underwent closure of transverse loop colostomy four months later.

The patient recovered well after each operation. Her symptoms of feculent vaginal discharge stopped following the defunctioning colostomy with no recurrence thus far. There was no recurrence of pelvic organ prolapse, up to six months after mesh removal.

Histological assessment of the excised sigmoid colon confirmed the fistulous tract with no evidence of a foreign body, suture or mesh within this tract.

## Discussion

A CVF is a rare complication following gynaecological surgery, with a CVF repair rate of 0.6 per 100,000 women [1]. It can cause distressing symptoms such as the passage of flatus, feculent material or discharge from the vagina [2,3] and result in the loss of patients’ quality of life.

Pelvic surgery is a known cause of pelvic organ fistulas, and women with previous hysterectomy are four times more likely to have pelvic organ fistula surgery compared to those without [4]. Intraoperative iatrogenic injury to the bowel and postoperative infections are the main factors involved in fistula formation after hysterectomy [5]. Difficult hysterectomies (for example, in cases of severe endometriosis or adhesions due to previous surgeries) may cause injuries to the rectum resulting in rectovaginal fistula formation [6]. Entry into the rectum during posterior colporrhaphy may also result in fistula formation. Placement of vaginal mesh is associated with a higher risk of fistula formation, and these are usually rectovaginal or vesicovaginal fistulas [1,7].

Although fistulas between the sigmoid and vagina are infrequent, they usually result from sigmoid diverticulitis and are most common in patients with a prior hysterectomy [8,9]. A study by Tancer and Veridiano reported that sigmoidovaginal fistulas are the commonest cologenital fistula caused by diverticulitis and usually present in women above the age of 50 years old with a history of a previous hysterectomy [8]. Similarly, a retrospective study conducted by Berger et al. which reviewed 19 cases of CVFs found that 79% of patients with CVFs had features of diverticulitis and 95% of patients with CVFs had a previous hysterectomy [9]. It has been postulated that a fistula develops between an inflamed segment of the sigmoid colon and the apical vaginal vault after repeated episodes of diverticulitis and abscess formation [2]. CVFs reported in these studies occurred in patients who underwent an abdominal hysterectomy, instead of vaginal surgery.

We reported an unusual case of a CVF complicating transvaginal prolapse surgery with the use of vaginal mesh.

The mechanism of CVF formation after vaginal hysterectomy and mesh-augmented pelvic floor repair in this case is not entirely clear. Our patient did not have any features of diverticulitis, despite CVFs being most commonly associated with diverticulitis. Although intraoperative trauma to the bowel could result in fistula formation, it is uncommon to cause direct injury to the sigmoid colon with vaginal surgery or mesh placement. Histology of the excised segment of the sigmoid also did not contain any mesh or suture material. One postulation could be that the development of postoperative vault hematoma or abscess formation caused local inflammation that subsequently led to CVF formation, although there were no clinical or radiological findings to confirm this hypothesis.

This case also highlights the possible mesh complications that could occur with the use of vaginal mesh. In this case, persistent faeces in the vagina as a result of the CVF may have resulted in poor wound healing and increased the risk of mesh extrusion.

The gold standard of treatment of CVFs is surgical repair, but the type of surgery performed depends on the surgeon’s judgement and patient’s condition [8]. CVFs are conventionally treated with staged surgery. Recent studies have suggested that resection of the diseased colon and primary anastomosis are also feasible and effective for most patients [9,10].

## Conclusions

This case demonstrates the CVF as a possible complication following gynaecology surgeries. Although uncommon, CVFs can cause distressing symptoms and significantly affect patients’ quality of life. This case also highlights the complexities of the management of CVFs, especially if other postoperative complications are present. Multidisciplinary management involving urogynaecologists and colorectal surgeons is important for optimising patient care and improving patient outcomes.
